# Psychosocial Factors Associated with Emotional Exhaustion Among Family Members of Individuals with Opioid Use Disorder: The Mediating Role of Perceived Social Support

**DOI:** 10.3390/healthcare14183055

**Published:** 2026-09-17

**Authors:** İbrahim Karakaya, Fırat Akdeniz

**Affiliations:** 1 Independent Researcher, Istanbul 34728, Türkiye; 2 Psychology Application and Research Center, Ankara Hacı Bayram Veli University, Ankara 06500, Türkiye

**Keywords:** opioid use disorder, emotional exhaustion, hopelessness, perceived social support, locus of control

## Abstract

**Objective:** The aim of this study was to examine the associations among hopelessness, emotional exhaustion, locus of control, and perceived social support among family members of individuals with opioid use disorder. **Method:** A total of 138 participants were included in the study from among the relatives of individuals seeking treatment for opioid use disorder. Participants completed the Beck Hopelessness Scale, the Maslach Burnout Inventory, the Rotter Internal–External Locus of Control Scale, the Multidimensional Scale of Perceived Social Support, and a sociodemographic information form. **Results:** A positive association was found between external locus of control and hopelessness and emotional exhaustion. Perceived social support partially mediated the association between external locus of control and emotional exhaustion. Significant differences in psychological outcomes were also observed across several demographic and socioeconomic characteristics, including gender, cohabitation with the patient, and income level. **Conclusions:** Among family members of individuals with opioid use disorder, perceived social support may represent an important psychosocial factor associated with emotional exhaustion. Strengthening social support mechanisms and addressing external locus of control may contribute to supporting the psychological well-being of family members.

## 1. Introduction

According to the World Health Organization (WHO), opioid use disorder (OUD) is a chronic psychiatric condition characterized by relapses and periods of recovery [1]. The 2024 World Drug Report published by the United Nations Office on Drugs and Crime (UNODC) indicates that opioids are among the most commonly used addictive substances worldwide, second only to cannabis [2]. Similarly, the 2019 Turkey report by the European Monitoring Centre for Drugs and Drug Addiction (EMCDDA) states that opioids are one of the three most commonly used illicit substances [3]. The same report indicates that individuals with OUD account for 58% of those seeking treatment for substance use disorders. Given these high rates, OUD represents an important public health issue that affects not only individuals with the disorder but also their family members [4,5,6,7,8].

Studies have demonstrated that substance use disorder has numerous negative effects on family members. From a psychological perspective, symptoms such as burnout and helplessness, depression, anxiety, and a decline in quality of life are frequently observed in families affected by substance use disorder [9,10,11,12,13]. Frequent relapses in substance use disorder, the chronic nature of the individual’s physical and mental health issues, and serious problems in academic, social, and economic spheres have been associated with psychological issues within the family, such as hopelessness, burnout, helplessness, feelings of failure, regret, denial, neglect, shame, guilt, and anger [13,14,15,16].

These psychological difficulties are further compounded by the psychosocial consequences associated with OUD. Criminal behavior, incarceration, unemployment, suicide attempts, and self-harm among individuals with OUD can exacerbate the psychological, social, and financial burden on families [8,17,18]. A study by Mattoo et al. (2013) found that families of individuals with OUD experience increased economic hardship and a significant disruption of family relationships and routines [19]. Similarly, in a study by Cicek et al. (2015) comparing individuals with OUD and their families to a control group, it was noted that families of individuals with addiction problems experienced significantly higher rates of physical illness, as well as psychological, social, financial, and environmental problems [20]. Taken together, these findings indicate that substance use disorder affects multiple domains of family functioning, extending beyond the psychological difficulties experienced by family members.

Beyond these direct and indirect consequences, substance use disorder also has broader implications for the family system. The family is affected, either directly or indirectly, at many stages of substance use disorder and plays a significant role in development, chronicity, treatment, and recovery [15,21]. Family structure, family relationships, and the physical and mental health of family members are key factors not only in the prevention of addiction but also in the treatment process for individuals with addiction problems [22,23,24]. Therefore, understanding the psychological, physical, and social difficulties experienced by family members affected by addiction is important for addressing the broader impact of substance use disorder.

Against this background, identifying psychological factors that may contribute to difficulties experienced by the family member is particularly important. Locus of control may represent one such factor in understanding psychological difficulties such as burnout and hopelessness among family members of individuals with OUD. Locus of control refers to a cognitive mechanism through which individuals attribute the situations they encounter to internal or external factors [25]. Accordingly, an external locus of control has been proposed to be associated with psychological distress [26]. In a study conducted in the addiction field, individuals with substance use disorder were found to attribute their subjective life events to an external locus of control more frequently [27]. However, the literature examining locus of control specifically among family members of individuals with addiction problems remains quite limited. Indirectly, some qualitative studies have shown that families attribute the addiction problem to external factors such as chance, fate, denial, or peer influence [28]. Based on this, it was hypothesized that external locus of control may be associated with psychological difficulties such as hopelessness and emotional exhaustion among family members.

On the other hand, perceived social support has an important function in coping with addiction for both the individual with the disorder and their relatives [15,21,29]. Furthermore, a reciprocal relationship has been proposed between perceived social support and depressive and anxiety symptoms among caregiving individuals [30]. Accordingly, when considered together with external locus of control, perceived social support was thought to be a mediating variable closely related to psychological problems such as hopelessness and emotional exhaustion among families.

Hopelessness, in turn, has also been closely linked to both external locus of control and reduced social support within the family caregiving literature, and it has itself been associated with burnout among relatives of individuals with substance use disorder [12]. It was therefore considered plausible that hopelessness might function, together with perceived social support, as an intervening factor in the association between external locus of control and emotional exhaustion.

Taken together, these findings indicate that psychological and social difficulties are prominent among family members of individuals with substance use disorder [8,13,14,15,16]. However, studies examining perceived social support together with locus of control, hopelessness, and emotional exhaustion among family members of individuals with OUD remain limited. In particular, the mechanisms through which perceived social support may link external locus of control to psychological difficulties such as hopelessness and emotional exhaustion have not been sufficiently clarified. Therefore, the present study aimed to examine whether perceived social support mediates the association between external locus of control and emotional exhaustion and whether perceived social support and hopelessness sequentially mediate this association among family members of individuals with OUD. Based on the reasoning above, the following hypotheses were tested:

**H1.**  *External locus of control is positively associated with hopelessness and emotional exhaustion and negatively associated with perceived social support.*

**H2.**  *Perceived social support is negatively associated with hopelessness and emotional exhaustion.*

**H3.**  *Perceived social support mediates the association between external locus of control and emotional exhaustion.*

**H4.**  *Perceived social support and hopelessness sequentially mediate the association between external locus of control and emotional exhaustion.*

## 2. Method

This study included 138 participants recruited on a voluntary basis from among the relatives of individuals who presented to the Psychiatry Outpatient Clinic at Özel Kapadokya Hospital for OUD treatment. Following psychiatric evaluation and diagnosis according to DSM-5 criteria, these patients received inpatient treatment for OUD. Because only one family member is permitted to accompany each patient during inpatient treatment, only one family member per patient was included in this study.

Although Özel Kapadokya Hospital operates as a private institution, individuals with lower socioeconomic status covered by Türkiye’s general health insurance system can also receive outpatient and inpatient treatment there. Accordingly, the family members of individuals with OUD included in the study varied considerably in socioeconomic characteristics, with the sample comprising both families with favorable economic conditions and families with limited economic means who were covered by public health insurance. Thus, although the sample was recruited from a single institution, it includes family members with diverse socioeconomic backgrounds rather than being restricted to higher-income families. Furthermore, logistical factors—such as the limited number of public hospitals offering inpatient OUD treatment in Turkey, high patient volume, confidentiality concerns, and the geographic concentration of public hospitals in major cities—often lead patients and families of varying socioeconomic backgrounds to seek treatment at private clinics such as the study site.

Participants were eligible if they were aged 18 years and older and did not have severe cognitive or neurodevelopmental impairments that could interfere with their ability to understand the study procedures or complete the assessment instrument. Family members who had advanced psychiatric or medical conditions, difficulty understanding the study instructions, were from the same family as another participant, had low motivation to complete the study materials, or were receiving treatment for another addictive disorder were not included in the study. Individuals under 18 years of age and those with neurological conditions such as dementia or intellectual disability were excluded.

Due to restrictions related to the COVID-19 pandemic, the time required to complete the data collection instruments, instances in which the accompanying relative was not a first-degree relative of the patient, and the difficulties experienced by families during the patient’s treatment process, data collection was conducted between 2021 and 2023. The scales and forms used in the study were administered by a psychologist working at the outpatient clinic. Exact figures for the number of relatives assessed for eligibility, excluded, or declining to participate were not systematically recorded.

Ethical approval for the study was obtained from Kapadokya University (27 May 2019, Decision No. 5). Participants completed the Beck Hopelessness Scale (BHS) [31], the Maslach Burnout Inventory (MBI) [32], the Multidimensional Scale of Perceived Social Support (MSPSS) [33], the Rotter Internal–External Locus of Control Scale (Rotter Scale) [25], and a sociodemographic information form developed by the researchers. A cross-sectional design was used to examine the associations among the study variables.

### 2.1. Data Collection Tools

Sociodemographic Information Form: The sociodemographic information form consists of 15 questions that assess participants’ demographic information—such as age, gender, marital status, and education level—as well as their relationship to the patient, cohabitation status, and income level.

Beck Hopelessness Scale: Developed by Beck and colleagues (1971) [31], the BHS is a self-report scale that assesses individuals’ loss of motivation, hope, feelings about the future, and expectations. The BHS, for which Durak and Palabıyıkoğlu [34] conducted a validity and reliability study in Turkish, consists of 20 questions and is scored on a scale of 0 to 1. A high score on the scale indicates a high level of hopelessness. Regarding the scale’s reliability, the Cronbach’s alpha value was determined to be 0.85.

Maslach Burnout Inventory: Developed by Maslach and Jackson (1981) [32], the MBI is a self-report scale consisting of 22 items organized into three subcategories: depersonalization, emotional exhaustion, and personal accomplishment. The MBI, for which Ergin [35] conducted a validity and reliability study in Turkish, is scored on a scale of 0–6, and each subscale is evaluated separately. High scores on the emotional exhaustion and depersonalization subscales, combined with a low score on the personal accomplishment subscale, indicate a high level of burnout. Regarding the scale’s reliability, the Cronbach’s alpha values for each subscale were determined as 0.83 for emotional exhaustion, 0.65 for depersonalization, and 0.72 for personal accomplishment. Although the MBI was originally developed to assess occupational burnout, it has also been used in the literature to examine burnout among family members of individuals with various chronic health and psychiatric conditions, including addictive disorders [36,37,38]. In the present study, the original item wording was used without modification; no items were reworded to specifically target the caregiving context.

Multidimensional Scale of Perceived Social Support: Developed by Zimet et al. (1988) [33], the MSPSS is a self-report scale consisting of three subcategories—family, friends, and significant other—and a total of 12 items. The MSPSS, whose validity and reliability were studied by Eker and Arkar [39], is scored on a scale of 1 to 7, and each subcategory is evaluated separately. A high score on the scale indicates a high level of social support. Regarding the scale’s reliability, the Cronbach’s alpha value was determined to be 0.89. Looking at the subcategories, the Cronbach’s alpha values were reported as 0.85 for family, 0.88 for friends, and 0.92 for significant other.

Rotter’s Internal–External Locus of Control Scale: Developed by Rotter (1966) [25], the Rotter Scale is a self-report scale consisting of 29 items that assesses the association between individuals’ life experiences and their internal or external locus of control. The Rotter Scale, for which validity and reliability studies were conducted by Dağ [40], is scored on a scale from 0 to 1, with higher scores on the scale being associated with an external locus of control. Regarding the scale’s reliability, the Cronbach’s alpha internal consistency coefficient was determined to be 0.70.

### 2.2. Data Analysis

The data obtained in this study were analyzed using the IBM SPSS Statistics 27 software package (International Business Machines, Turkish Limited Company, İstanbul, Türkiye). To describe the participants’ demographic characteristics, frequency (n) and percentage (%) values were calculated; for age, the mean (M), standard deviation (SD), minimum, and maximum values were calculated. For scale scores, descriptive statistics, skewness and kurtosis coefficients, and Cronbach’s alpha internal consistency coefficients were examined. Associations between variables were assessed using Pearson’s product–moment correlation analysis. The mediating roles of perceived social support and hopelessness in the association between external locus of control and emotional exhaustion were tested using regression equations consistent with the PROCESS Model 6 logic and 5000 bootstrap samples. In the mediation analysis, age, gender, living with the patient, employment status, and income level were controlled for as covariates. Income level was represented by two dummy variables, and the group earning above the minimum wage was taken as the reference category. Prior to regression, the normality and homoscedasticity of the residuals, as well as the independence of the error terms and the assumption of no multicollinearity, were checked. For demographic group comparisons, the independent-samples *t*-test or Welch *t*-test was used for variables with two groups; one-way ANOVA or Welch ANOVA was used for variables with three or more groups. For significant ANOVA results, Tukey or Games–Howell post hoc tests were used; Cohen’s d was reported for *t*-tests, and η^2^ was reported for ANOVA results. Given the large number of demographic subgroup comparisons conducted, these analyses should be considered exploratory in nature. No correction for multiple comparisons was applied; therefore, the increased risk of false-positive findings should be considered when interpreting these results. In all analyses, the level of significance was set at *p* < 0.05. A sensitivity power analysis was subsequently conducted for the regression models used in the mediation analysis, calculated using the noncentral F-distribution for multiple regression with the covariates included [41]. Given the achieved sample size (N = 138) and the degrees of freedom of each model, the analysis indicated that partial effects as small as f^2^ ≈ 0.06 (80% power, α = 0.05), corresponding to a partial correlation of approximately 0.24, were detectable.

## 3. Results

Upon examining the findings, it was determined that the participants’ average age was M = 48.47 (SD = 12.01; min = 25, max = 65). In terms of gender distribution, 64.49% of the participants (n = 89) were female, and 35.51% (n = 49) were male. Full demographic characteristics are presented in Table 1.

The results of the analyses in Table 2 showed that the skewness values for the variables ranged from −0.71 to 0.83, while the kurtosis values ranged from −1.19 to −0.22. The fact that all skewness and kurtosis coefficients fall within the ±2 limits indicates that the scale scores are sufficiently close to a normal distribution and that the necessary assumption for applying parametric tests is met [42].

The Cronbach’s alpha (α) internal consistency coefficients, calculated to examine the reliability of the measurement instruments, were found to range from 0.79 to 0.90 (Table 2). The fact that all coefficients are above 0.70 indicates that the scales and subscales used in the study possess acceptable to high levels of internal consistency [43].

An analysis of the Table 3 revealed positive correlations between emotional exhaustion and depersonalization (r = 0.56, *p* < 0.001), hopelessness (r = 0.51, *p* < 0.001), and an external locus of control (r = 0.44, *p* < 0.001), and negative significant correlations were found between emotional exhaustion and perceived social support (r = −0.53, *p* < 0.001) and personal accomplishment (r = −0.33, *p* < 0.001). Significant negative correlations were found between perceived social support and hopelessness (r = −0.53, *p* < 0.001) and an external locus of control (r = −0.40, *p* < 0.001). In addition, a moderate and significant positive association was found between hopelessness and an external locus of control (r = 0.49, *p* < 0.001). Overall, the observed bivariate associations were consistent with the directions hypothesized in the Introduction.

According to the Table 4, the assumptions were examined prior to the regression analysis. The Durbin–Watson statistic of 2.30 indicated that the error terms were independent. Tolerance values ranged from 0.44 to 0.90, and VIF values ranged from 1.11 to 2.30; no multicollinearity was found. It was determined that the residuals met the assumption of normal distribution (Shapiro–Wilk *p* = 0.751) and that the error variances were homogeneous (Breusch–Pagan *p* = 0.290).

When controlling for covariates, the total effect of external locus of control on emotional exhaustion was positive and significant (B = 0.59, β = 0.41, t(130) = 5.32, *p* < 0.001, 95% CI [0.37, 0.82]). The total effect model explained 39.88% of the variance in emotional exhaustion (R^2^ = 0.40, F(7, 130) = 12.32, *p* < 0.001). External locus of control negatively predicted perceived social support (B = −1.11, β = −0.33, t(130) = −3.88, *p* < 0.001), and this model explained 27.07% of the variance in perceived social support scores (R^2^ = 0.27, F(7, 130) = 6.89, *p* < 0.001).

The covariate-adjusted model for the prediction of hopelessness was significant (R^2^ = 0.54, F(8, 129) = 18.85, *p* < 0.001). External locus of control predicted hopelessness positively (B = 0.15, β = 0.19, t(129) = 2.68, *p* = 0.008), while perceived social support predicted it negatively (B = −0.06, β = −0.28, t(129) = −3.99, *p* < 0.001).

The direct effect model for predicting emotional exhaustion was significant and explained 48.17% of the total variance (R^2^ = 0.48, F(9, 128) = 13.22, *p* < 0.001). External locus of control (B = 0.40, β = 0.28, t(128) = 3.50, *p* < 0.001), hopelessness (B = 0.36, β = 0.19, t(128) = 2.03, *p* = 0.044), and perceived social support (B = −0.11, β = −0.25, t(128) = −3.14, *p* = 0.002) were significant predictors of emotional exhaustion. When mediating variables were included in the model, the direct effect of external locus of control decreased but remained significant.

Upon examining the bootstrap results in Table 5, it was determined that the indirect effect of perceived social support on the association between external locus of control and emotional exhaustion was significant (effect = 0.12, 95% CI [0.04, 0.24]). In contrast, the indirect effect mediated solely through hopelessness (effect = 0.05, 95% CI [−0.01, 0.14]) and the serial indirect effect in which perceived social support and hopelessness were sequentially involved (effect = 0.03, 95% CI [−0.003, 0.065]) were not significant due to confidence intervals that included zero. The total indirect effect, however, was significant (effect = 0.20, 95% CI [0.08, 0.35]).

The results indicated that, when demographic covariates were controlled for, perceived social support played a partial mediating role on its own in the association between an external locus of control and emotional exhaustion. The mediating effect of hopelessness alone and the serial indirect effect from perceived social support to hopelessness were not supported. Since the findings are based on cross-sectional data, they should not be interpreted as evidence of temporal or experimental causality.

**Figure 1:**
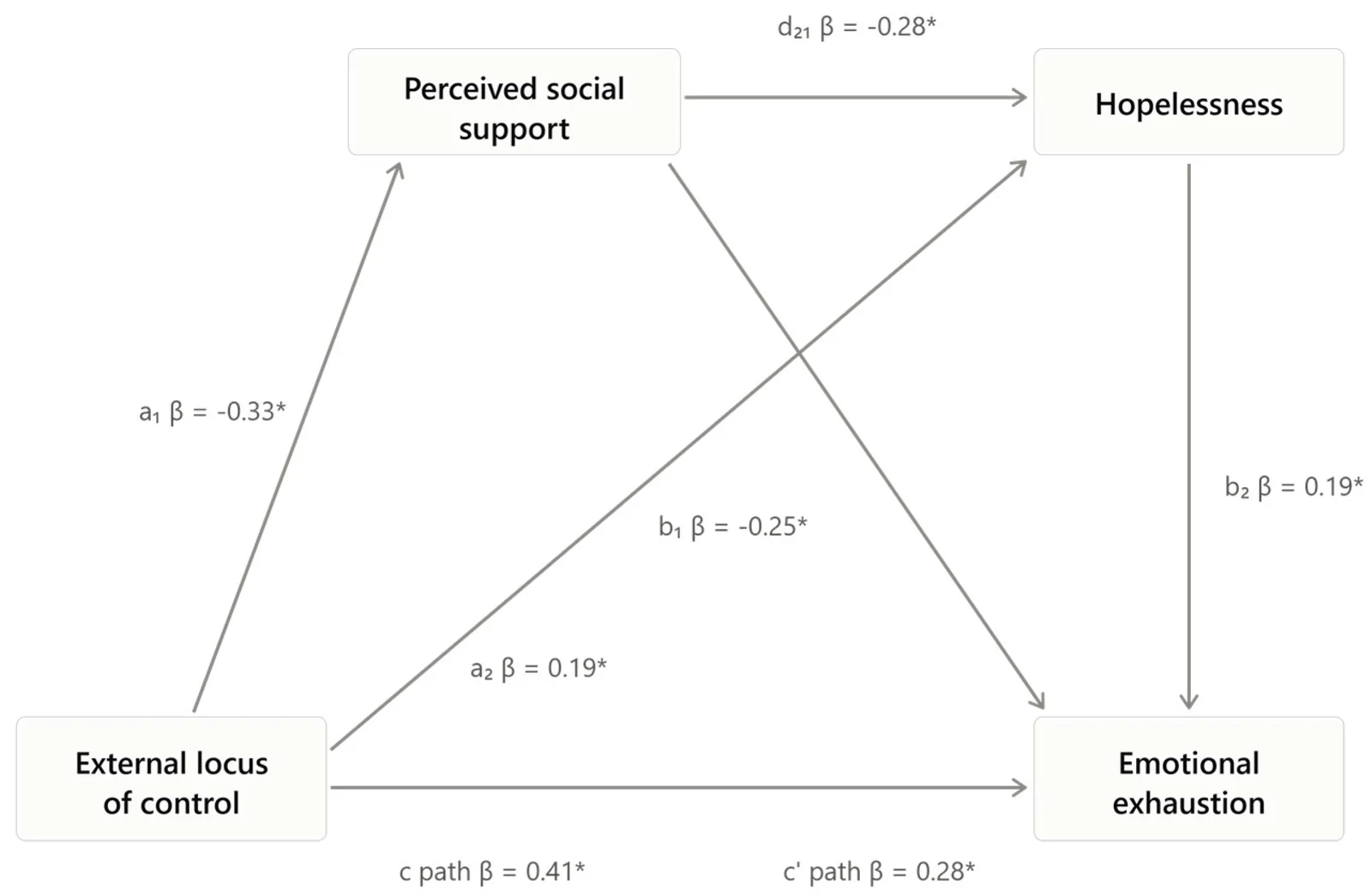
Standardized Path Coefficients for the Covariate-Adjusted Serial Mediation Model. Note. Standardized regression coefficients (β) are reported. * *p* < 0.05.

Standardized path coefficients for the covariate-adjusted serial mediation model are shown in Figure 1.

The following demographic group comparisons (Table 6) should be interpreted as exploratory, given the number of comparisons conducted and the absence of a multiple-comparisons correction. Findings related to gender, cohabitation with the patient, and income level are highlighted below as they showed the most consistent associations across multiple outcome variables and are most directly relevant to the study’s objectives; the remaining comparisons are reported in full in Table 6 for completeness. An examination of Table 6 revealed that emotional exhaustion and hopelessness scores differed significantly by gender. Women’s emotional exhaustion scores (M = 16.36, SD = 8.57) were significantly higher than men’s scores (M = 12.55, SD = 8.59), t(136) = −2.50, *p* = 0.014, d = −0.44. Similarly, women’s hopelessness scores (M = 6.66, SD = 4.70) were found to be higher than men’s scores (M = 4.69, SD = 4.23), t(136) = −2.44, *p* = 0.016, d = −0.43.

Based on living arrangements with the patient, participants living with the patient had significantly higher emotional exhaustion scores (M = 16.66, SD = 8.41) than those not living with the patient (M = 9.07, SD = 7.29), t(136) = −4.49, *p* < 0.001, d = −0.93. The depersonalization scores of participants living with the patient (M = 5.92, SD = 4.83) were also found to be higher than those of participants not living with the patient (M = 2.83, SD = 3.18), t(70.10) = −4.14, *p* < 0.001, d = −0.68. The perceived social support scores of those living together (M = 54.72, SD = 20.74) were lower than those of those not living together (M = 63.87, SD = 17.17), t(54.80) = 2.46, *p* = 0.017, d = 0.46. In addition, the hopelessness scores of those living together (M = 6.45, SD = 4.66) were found to be higher than those of those not living together (M = 4.20, SD = 4.09), t(136) = −2.40, *p* = 0.018, d = −0.50.

By marital status, a significant difference was found only in hopelessness scores, F(2, 45.50) = 7.12, *p* = 0.002, η^2^ = 0.07. Married participants’ hopelessness scores (M = 6.81, SD = 4.89) were higher than those of single (M = 4.00, SD = 3.39) and divorced/widowed participants (M = 4.17, SD = 3.30).

In terms of employment status, the hopelessness scores of unemployed participants (M = 6.93, SD = 4.90) were found to be higher than those of employed participants (M = 4.81, SD = 4.00), t(135.90) = −2.80, *p* = 0.006, d = −0.47. The external locus of control scores for unemployed participants (M = 12.43, SD = 6.05) were also significantly higher than those of employed participants (M = 8.25, SD = 5.29), t(136) = −4.27, *p* < 0.001, d = −0.73.

By age group, a significant difference was found only in hopelessness scores, F(2, 88.11) = 6.55, *p* = 0.002, η^2^ = 0.07. The hopelessness scores of the 40–54 age group (M = 6.70, SD = 4.85) and the 55 and older age group (M = 6.84, SD = 4.97) were higher than those of the 25–39 age group (M = 4.16, SD = 3.37).

A significant difference was found in emotional exhaustion scores based on the relationship to the patient, F(3, 134) = 6.53, *p* < 0.001, η^2^ = 0.13. The emotional exhaustion scores of participants whose relationship to the patient was spouse (M = 21.38, SD = 7.98) were higher than those of participants whose relationship to the patient was father (M = 12.81, SD = 8.56), mother (M = 15.39, SD = 8.15), or sibling (M = 11.55, SD = 8.39). It should be noted that the spouse subgroup was relatively small (n = 21) compared to the other relationship categories; therefore, this finding should be interpreted with some caution and considered preliminary pending replication in larger samples. Hopelessness scores also differed significantly according to the degree of closeness to the patient, F(3, 134) = 4.32, *p* = 0.006, η^2^ = 0.09. The hopelessness scores of mothers (M = 7.31, SD = 4.77) were higher than those of siblings (M = 3.90, SD = 3.77).

A significant difference was found in perceived social support scores based on educational level, F(3, 57.96) = 3.57, *p* = 0.019, η^2^ = 0.07. University graduates had higher social support scores (M = 66.83, SD = 17.73) than primary school graduates (M = 54.21, SD = 18.77) and middle school graduates (M = 50.23, SD = 19.80). A significant and substantial difference in hopelessness scores was found based on educational level, F(3, 134) = 9.25, *p* < 0.001, η^2^ = 0.17. The hopelessness scores of primary school graduates (M = 8.02, SD = 4.71) were higher than those of high school graduates (M = 4.78, SD = 3.97) and university graduates (M = 2.91, SD = 3.15). External locus of control scores also differed significantly by educational level, F(3, 134) = 5.22, *p* = 0.002, η^2^ = 0.10. The external locus of control scores for primary school graduates (M = 12.73, SD = 6.23) were higher than those of high school graduates (M = 8.95, SD = 5.32) and university graduates (M = 7.87, SD = 5.35).

Significant and substantial differences were found in emotional exhaustion scores by income level, F(2, 135) = 11.02, *p* < 0.001, η^2^ = 0.14. The emotional exhaustion scores of those with incomes below the minimum wage (M = 18.33, SD = 8.52) were higher than those of individuals with incomes above the minimum wage (M = 11.10, SD = 8.29). Perceived social support scores also differed significantly by income level, F(2, 135) = 12.20, *p* < 0.001, η^2^ = 0.15. The social support scores of those with incomes above the minimum wage (M = 66.60, SD = 17.22) were higher than those of participants with incomes at the minimum wage level (M = 54.48, SD = 21.18) and those with incomes below the minimum wage (M = 49.20, SD = 19.14). Significant differences in hopelessness scores were found across income levels, F(2, 61.06) = 41.34, *p* < 0.001, η^2^ = 0.35. The hopelessness scores of those with incomes below the minimum wage (M = 8.69, SD = 4.38) were higher than those of individuals earning at the minimum wage level (M = 6.16, SD = 4.03) and those earning above the minimum wage (M = 2.67, SD = 2.68). The scores of the group at the minimum wage level were also higher than those of the group with income above the minimum wage. External locus of control scores differed significantly and substantially by income level, F(2, 135) = 14.36, *p* < 0.001, η^2^ = 0.18. The external locus of control scores for those with incomes below the minimum wage (M = 13.16, SD = 5.95) were higher than those for individuals with incomes above the minimum wage (M = 7.56, SD = 5.29).

## 4. Discussion

This study examined the associations between burnout, hopelessness, perceived social support, and locus of control among family members of individuals with OUD. The findings were generally consistent with the first three hypotheses outlined above (H1–H3), whereas the sequential mediation proposed in H4 was not supported.

First, emotional exhaustion among family members was positively associated with depersonalization, hopelessness, and external locus of control. Similarly, Şenormancı et al. [12] reported that family members of individuals with substance use disorder were at greater risk of developing burnout, hopelessness, and depression compared to a control group. Recent studies, however, suggest that these psychological problems among family members are associated with cognitive, emotional, financial, and social difficulties related to the chronic nature of addiction [5,6]. The severe prognosis of OUD, its frequent relapses, and its legal, financial, and social consequences may be associated with a weakened sense of control among family members, as well as greater social isolation and emotional exhaustion [44,45]. In the present study, it was also found that symptoms of hopelessness and emotional exhaustion were positively associated with an external locus of control and negatively associated with perceived social support. Furthermore, mediation analyses indicated that perceived social support partially mediated the association between external locus of control and emotional exhaustion. In addition, higher perceived social support and personal accomplishment were associated with lower emotional exhaustion. Taken together, these findings suggest that perceived social support may represent an important intervening factor in the association between external locus of control and emotional exhaustion among family members of individuals with OUD [45,46].

OUD is a psychiatric disorder that affects not only individuals with the disorder but also family systems. In this study, family members living with individuals with OUD had higher emotional exhaustion, depersonalization, and hopelessness scores and lower perceived social support than those not living with the patient. In particular, female family members were found to have higher emotional exhaustion and hopelessness scores compared to men. Spouses reported more symptoms of emotional exhaustion than other family members, although this finding is based on a relatively small subgroup (n = 21) and should be interpreted with corresponding caution. In a study by Shamsaei et al. [11], it was noted that spouses experienced more problems in the areas of anxiety and interpersonal sensitivity compared to other family members; however, no significant difference was found between gender and psychological problems.

Similarly, in a study by Korkmaz et al. [16] conducted with the spouses of male patients with alcohol use disorder, it was observed that 80% experienced a decline in social relationships, 66% experienced irritability, 46% experienced tension, 29% experienced sadness, and 20% experienced feelings of weariness and anger. Studies in the literature indicate that the burden of caregiving is closely associated with depression and anxiety, as well as impairments in quality of life, among women [10,23,47]. These findings, together with previous evidence, suggest that gender roles may contribute to differences in caregiving burden and psychological distress among women. These findings highlight the potential value of tailored, structured, and accessible interventions that consider gender-related caregiving demands and aim to strengthen the psychological and social resources of family members [5].

Finally, significant findings were observed between the socioeconomic characteristics of family members—such as age, income level, education, and employment status—and certain variables. Hopelessness differed across age groups, with higher scores among participants aged 40–54 and 55 years or older than among those aged 25–39 years. Higher educational level was associated with lower hopelessness and external locus of control scores and higher perceived social support. Lower income levels were associated with higher hopelessness, emotional exhaustion, and external locus of control, as well as lower perceived social support. Similarly, it was determined that hopelessness and external locus of control scores were higher among family members not actively engaged in the workforce compared to those who were employed. In a study by Mattoo et al. [19], it was reported that the income level of family members of patients and residing in rural areas were associated with the burden of caregiving. However, unlike the findings of that study, no significant association was found between age, educational level, and occupational status and the burden of caregiving. In conclusion, socioeconomic conditions among family members of individuals with substance use disorders were significantly associated with several psychological and social outcomes, including hopelessness, emotional exhaustion, and perceived social support.

## 5. Conclusions

In this study, hopelessness and external locus of control were positively associated with emotional exhaustion among family members of individuals with OUD. According to mediation analyses, perceived social support partially mediated the association between external locus of control and emotional exhaustion. Although hopelessness was associated with emotional exhaustion, it did not play a significant mediating role; thus, the serial mediation hypothesis (H4) was not supported. Furthermore, women, spouses, those living with the patient, and those with lower socioeconomic resources showed greater psychological vulnerability across several study outcomes. These findings suggest that social support, socioeconomic conditions, and locus of control may be relevant considerations for future research and for the development of psychosocial approaches aimed at supporting family members of individuals with OUD.

### Limitations and Recommendations

First, this study included family members of individuals with OUD who sought treatment at the psychiatry outpatient clinic of a single private hospital in Turkey. Therefore, the generalizability of the findings to family members of individuals with OUD in other clinical, geographical, and cultural settings may be limited. Future comparative studies focusing on family members of individuals with different substance use disorders could contribute to the literature.

Additionally, although the MBI has precedent in the literature for use with family caregivers, it was originally developed and validated for occupational burnout. Its psychometric properties have not been specifically validated for family members of individuals with substance use disorders in the Turkish context, and this should be considered when interpreting the burnout-related findings of this study.

Second, although 138 family members participated in this study, the sample sizes of some demographic subgroups were relatively small, which may have limited the statistical power of subgroup comparisons. Studies with larger and more diverse samples are needed to further examine psychological outcomes among family members of individuals with OUD. A formal a priori power analysis was not conducted prior to data collection. A post hoc sensitivity analysis indicated that the achieved sample size was sufficient to detect partial effects of f^2^ ≈ 0.06 or larger (80% power, α = 0.05) for the individual paths in the mediation model. Most paths in the model exceeded this threshold; however, the association between hopelessness and emotional exhaustion (f^2^ = 0.03) and, to a lesser extent, the path from external locus of control to hopelessness (f^2^ = 0.06) approached the lower bound of detectable effects, suggesting these specific associations should be interpreted with some caution and re-examined in future studies with larger samples. Similarly, precise recruitment figures—including the number of relatives assessed for eligibility, excluded, or declining participation—were not systematically documented, which limits the ability to evaluate potential selection bias in the final sample.

Third, the cross-sectional design of the study precludes conclusions regarding temporal or causal relationships among external locus of control, perceived social support, hopelessness, and emotional exhaustion. Therefore, the mediation findings should be interpreted as statistical indirect associations rather than evidence of causal mechanisms. Longitudinal studies are needed to clarify the temporal relationships among these variables. Additionally, data collection took place between 2021 and 2023, partly overlapping with the COVID-19 pandemic period. Locus of control is generally considered a relatively stable, trait-like disposition that is less susceptible to short-term societal change, and findings related to this construct are likely to remain broadly applicable. However, perceived social support and emotional exhaustion may be more sensitive to context-dependent factors, such as prevailing economic conditions, healthcare access, or public health circumstances and may therefore be more susceptible to change over time. Findings related to these constructs should accordingly be interpreted with somewhat greater caution regarding their applicability to current circumstances.

Finally, given the important role of families in the treatment and recovery processes of substance use disorders, further research should examine the psychosocial well-being and support needs of family members affected by OUD.

## Figures and Tables

**Table 1:** Distribution of Participants’ Demographic Characteristics.

	Category	n	%
Age			
	25–39	43	31.16
	40–54	44	31.88
	55 and above	51	36.96
Gender			
	Male	49	35.51
	Female	89	64.49
Living with the patient			
	No	30	21.74
	Yes	108	78.26
Relationship to the patient			
	Father	26	18.84
	Mother	62	44.93
	Spouse	21	15.22
	Sibling	29	21.01
Education level			
	Primary school graduate	56	40.58
	Middle school graduate	22	15.94
	High school graduate	37	26.81
	University graduate	23	16.67
Marital status			
	Single	20	14.49
	Married	95	68.84
	Divorced/Widowed	23	16.67
Employment status			
	Employed	63	45.65
	Unemployed	75	54.35
Income level			
	Below minimum wage	61	44.20
	Minimum wage	25	18.12
	Above minimum wage	52	37.68

Note. N = 138. Percentages may not sum to 100% due to rounding. “Minimum wage” refers to the national minimum wage in Turkey applicable at the time of data collection (2021–2023).

**Table 2:** Descriptive Statistics, Normality, and Reliability Results for the Scales.

	n	Min.	Max.	M	SD	Skewness	Kurtosis	α
Emotional Exhaustion	138	0	35	15.01	8.74	0.14	−0.80	0.89
Depersonalization	138	0	17	5.25	4.69	0.83	−0.42	0.79
Personal Accomplishment	138	2	32	21.69	6.93	−0.71	−0.22	0.86
Perceived Social Support	138	15	84	56.71	20.31	−0.33	−1.19	0.90
Hopelessness	138	0	18	5.96	4.62	0.71	−0.45	0.88
External Locus of Control	138	0	23	10.52	6.07	0.16	−0.92	0.90

Note. M = mean; SD = standard deviation; α = Cronbach’s alpha internal consistency coefficient. Skewness and kurtosis values within ±2 indicate an acceptable approximation to a normal distribution (Kline, 2015) [42].

**Table 3:** Correlation Results Among Variables.

Variable	1	2	3	4	5	6
1. Emotional Exhaustion	—					
2. Depersonalization	0.56 ***	—				
3. Personal Accomplishment	−0.33 ***	−0.21 *	—			
4. Perceived Social Support	−0.53 ***	−0.40 ***	0.44 ***	—		
5. Hopelessness	0.51 ***	0.29 ***	−0.41 ***	−0.53 ***	—	
6. External Locus of Control	0.44 ***	0.30 ***	−0.27 **	−0.40 ***	0.49 ***	—

Note. * *p* < 0.05. ** *p* < 0.01. *** *p* < 0.001. Correlation coefficients were calculated using Pearson’s product–moment method.

**Table 4:** Regression Results for the Serial Mediation Model with Covariates Controlled.

	Predictor	B	SE	Β	t	*p*	95% CI [LL, UL]
Emotional Exhaustion (total effect)	External Locus of Control	0.59	0.11	0.41	5.32	<0.001	[0.37, 0.82]
Perceived Social Support	External Locus of Control	−1.11	0.29	−0.33	−3.88	<0.001	[−1.68, −0.54]
Hopelessness	External Locus of Control	0.15	0.05	0.19	2.68	0.008	[0.04, 0.26]
	Perceived Social Support	−0.06	0.02	−0.28	−3.99	<0.001	[−0.10, −0.03]
Emotional Exhaustion (direct effect model)	External Locus of Control	0.40	0.11	0.28	3.50	<0.001	[0.17, 0.62]
	Perceived Social Support	−0.11	0.03	−0.25	−3.14	0.002	[−0.17, −0.04]
	Hopelessness	0.36	0.18	0.19	2.03	0.044	[0.01, 0.71]

Note. SE = standard error; CI = confidence interval; LL = lower limit; UL = upper limit. Age, gender, living with the patient, employment status, and income level were controlled for in all equations. Total effect model: R^2^ = 0.40, F(7, 130) = 12.32, *p* < 0.001; perceived social support: R^2^ = 0.27, F(7, 130) = 6.89, *p* < 0.001; hopelessness: R^2^ = 0.54, F(8, 129) = 18.85, *p* < 0.001; direct effect model: R^2^ = 0.48, F(9, 128) = 13.22, *p* < 0.001.

**Table 5:** Bootstrap Indirect Effects with Covariates Controlled.

	Unstandardized Indirect Effect	BootSE	BootLLCI	BootULCI
External Locus of Control → Perceived Social Support → Emotional Exhaustion	0.12	0.05	0.04	0.24
External Locus of Control → Hopelessness → Emotional Exhaustion	0.05	0.04	−0.01	0.14
External Locus of Control → Perceived Social Support → Hopelessness → Emotional Exhaustion	0.03	0.02	−0.003	0.065
Total indirect effect	0.20	0.07	0.08	0.35

Note. Age, gender, living with the patient, employment status, and income level were controlled for. Indirect effects were calculated using unstandardized coefficients. BootSE = bootstrap standard error; BootLLCI and BootULCI = lower and upper limits of the bootstrap 95% confidence interval. The number of bootstrap samples was 5000.

**Table 6:** Comparison of Scale Scores by Demographic Variables.

	Group	N	Emotional Exhaustion M (SD)	Depersonalization M (SD)	Personal Accomplishment M (SD)	Perceived Social Support M (SD)	Hopelessness M (SD)	External Locus of Control M (SD)
Gender	Male	49	12.55 (8.59)	4.96 (4.97)	21.57 (7.43)	59.24 (20.89)	4.69 (4.23)	9.16 (5.59)
	Female	89	16.36 (8.57)	5.40 (4.54)	21.75 (6.68)	55.31 (19.97)	6.66 (4.70)	11.27 (6.22)
	Test and *p*		t = −2.50, *p* = 0.014 *; d = −0.44	t = −0.53, *p* = 0.595; d = −0.09	t = −0.15, *p* = 0.884; d = −0.03	t = 1.09, *p* = 0.278; d = 0.19	t = −2.44, *p* = 0.016 *; d = −0.43	t = −1.97, *p* = 0.051; d = −0.35
Living with the patient	No	30	9.07 (7.29)	2.83 (3.18)	23.67 (6.27)	63.87 (17.17)	4.20 (4.09)	10.47 (4.78)
	Yes	108	16.66 (8.41)	5.92 (4.83)	21.14 (7.03)	54.72 (20.74)	6.45 (4.66)	10.54 (6.40)
	Test and *p*		t = −4.49, *p* < 0.001 *†; d = −0.93	Welch t = −4.14, *p* < 0.001 *†; d = −0.68	t = 1.78, *p* = 0.077; d = 0.37	Welch t = 2.46, *p* = 0.017 *; d = 0.46	t = −2.40, *p* = 0.018 *; d = −0.50	Welch t = −0.07, *p* = 0.948; d = −0.01
Marital status	Single	20	13.10 (8.33)	5.35 (5.38)	23.60 (7.16)	58.65 (17.44)	4.00 (3.39)	8.80 (4.74)
	Married	95	15.48 (8.94)	5.20 (4.62)	20.96 (7.21)	55.09 (21.48)	6.81 (4.89)	11.25 (6.51)
	Divorced/Widowed	23	14.70 (8.36)	5.35 (4.55)	23.04 (4.98)	61.70 (17.15)	4.17 (3.30)	9.00 (4.61)
	Test and *p*		F = 0.63, *p* = 0.535; η^2^ = 0.01	F = 0.01, *p* = 0.985; η^2^ = 0.00	F = 1.75, *p* = 0.178; η^2^ = 0.03	Welch F = 1.29, *p* = 0.285; η^2^ = 0.02	Welch F = 7.12, *p* = 0.002 *; η^2^ = 0.07	Welch F = 2.84, *p* = 0.069; η^2^ = 0.03
Employment status	Employed	63	14.90 (8.81)	5.43 (4.89)	22.06 (7.22)	58.02 (19.59)	4.81 (4.00)	8.25 (5.29)
	Unemployed	75	15.09 (8.74)	5.09 (4.54)	21.37 (6.71)	55.61 (20.96)	6.93 (4.90)	12.43 (6.05)
	Test and *p*		t = −0.13, *p* = 0.900; d = −0.02	t = 0.42, *p* = 0.677; d = 0.07	t = 0.58, *p* = 0.562; d = 0.10	t = 0.69, *p* = 0.491; d = 0.12	Welch t = −2.80, *p* = 0.006 *; d = −0.47	t = −4.27, *p* < 0.001 *†; d = −0.73
Age group	25–39	43	15.07 (9.24)	5.63 (4.81)	22.14 (6.69)	59.37 (19.61)	4.16 (3.37)	9.09 (5.79)
	40–54	44	15.39 (8.74)	5.52 (4.86)	20.61 (7.08)	53.52 (19.73)	6.70 (4.85)	11.16 (6.46)
	55 and above	51	14.63 (8.46)	4.69 (4.47)	22.24 (7.02)	57.22 (21.38)	6.84 (4.97)	11.18 (5.86)
	Test and *p*		F = 0.09, *p* = 0.914; η^2^ = 0.00	F = 0.58, *p* = 0.562; η^2^ = 0.01	F = 0.78, *p* = 0.462; η^2^ = 0.01	F = 0.93, *p* = 0.399; η^2^ = 0.01	Welch F = 6.55, *p* = 0.002 *; η^2^ = 0.07	F = 1.75, *p* = 0.178; η^2^ = 0.03
Relationship to the patient	Father	26	12.81 (8.56)	4.77 (4.67)	21.50 (7.86)	61.69 (19.97)	5.04 (4.63)	9.77 (6.09)
	Mother	62	15.39 (8.15)	4.85 (4.33)	21.84 (6.74)	55.03 (20.10)	7.31 (4.77)	11.55 (6.13)
	Spouse	21	21.38 (7.98)	7.38 (5.36)	20.57 (6.99)	49.48 (22.13)	6.00 (4.15)	11.10 (6.64)
	Sibling	29	11.55 (8.39)	4.97 (4.75)	22.34 (6.67)	61.07 (18.55)	3.90 (3.77)	8.59 (5.17)
	Test and *p*		F = 6.53, *p* < 0.001 *†; η^2^ = 0.13	F = 1.75, *p* = 0.160; η^2^ = 0.04	F = 0.28, *p* = 0.840; η^2^ = 0.01	F = 2.04, *p* = 0.111; η^2^ = 0.04	F = 4.32, *p* = 0.006 *; η^2^ = 0.09	F = 1.80, *p* = 0.150; η^2^ = 0.04
Education level	Primary school	56	16.88 (8.79)	4.93 (4.33)	21.34 (6.71)	54.21 (18.77)	8.02 (4.71)	12.73 (6.23)
	Middle school	22	15.05 (8.67)	6.77 (4.99)	20.86 (5.15)	50.23 (19.80)	5.91 (4.46)	10.32 (5.90)
	High school	37	13.86 (8.01)	5.08 (4.86)	23.19 (7.58)	58.05 (22.55)	4.78 (3.97)	8.95 (5.32)
	University	23	12.26 (9.31)	4.83 (4.99)	20.91 (7.85)	66.83 (17.73)	2.91 (3.15)	7.87 (5.35)
	Test and *p*		F = 1.86, *p* = 0.140; η^2^ = 0.04	F = 0.94, *p* = 0.424; η^2^ = 0.02	F = 0.82, *p* = 0.484; η^2^ = 0.02	Welch F = 3.57, *p* = 0.019 *; η^2^ = 0.07	F = 9.25, *p* < 0.001 *†; η^2^ = 0.17	F = 5.22, *p* = 0.002 *; η^2^ = 0.10
Income level	Below minimum wage	61	18.33 (8.52)	6.33 (4.85)	20.34 (7.11)	49.20 (19.14)	8.69 (4.38)	13.16 (5.95)
	Minimum wage	25	15.04 (6.88)	4.12 (4.24)	21.56 (6.64)	54.48 (21.18)	6.16 (4.03)	10.24 (5.04)
	Above minimum wage	52	11.10 (8.29)	4.52 (4.52)	23.33 (6.62)	66.60 (17.22)	2.67 (2.68)	7.56 (5.29)
	Test and *p*		F = 11.02, *p* < 0.001 *†; η^2^ = 0.14	F = 3.06, *p* = 0.050; η^2^ = 0.04	F = 2.67, *p* = 0.073; η^2^ = 0.04	F = 12.20, *p* < 0.001 *†; η^2^ = 0.15	Welch F = 41.34, *p* < 0.001 *†; η^2^ = 0.35	F = 14.36, *p* < 0.001 *†; η^2^ = 0.18

Note. M = mean; SD = standard deviation. * *p* < 0.05. Independent-samples *t*-test or, where indicated by †, Welch’s *t*-test was used for two-group comparisons where the homogeneity of variance assumption was violated; one-way ANOVA or, where indicated by †, Welch’s ANOVA and Games–Howell post hoc tests were used for multi-group comparisons.

## Data Availability

The data presented in this study are available upon reasonable request from the corresponding author. The data cannot be publicly shared due to ethical and privacy-related restrictions.
